# Molecular Survey of Vector-Borne Pathogens in Ticks, Sheep Keds, and Domestic Animals from Ngawa, Southwest China

**DOI:** 10.3390/pathogens11050606

**Published:** 2022-05-22

**Authors:** Miao Lu, Junhua Tian, Hongqing Zhao, Hai Jiang, Xincheng Qin, Wen Wang, Kun Li

**Affiliations:** 1 National Institute for Communicable Disease Control and Prevention, Chinese Center for Disease Control and Prevention, Changping Liuzi 5 C, Beijing 102206, China; lumiao@icdc.cn (M.L.); zhaohongqing@icdc.cn (H.Z.); jianghai@icdc.cn (H.J.); qinxincheng@icdc.cn (X.Q.); wangwen@icdc.cn (W.W.); 2 Wuhan Center for Disease Control and Prevention, Wuhan 430024, China; tianjunhua1980@163.com

**Keywords:** SFG *Rickettsia*, *Anaplasma bovis*, *Coxiella*, Ngawa, Southwest China

## Abstract

Vector-borne pathogens are mainly transmitted by blood-feeding arthropods such as ticks, mosquitoes, fleas, lice, mites, etc. They pose a significant threat to animal and human health due to their worldwide distribution. Although much work has been performed on these pathogens, some neglected areas and undiscovered pathogens are still to be further researched. In this study, ticks (*Haemaphysalis qinghaiensis*), sheep keds (*Melophagus ovinus*), and blood samples from yaks and goats were collected in Ngawa Tibetan and Qiang Autonomous Prefecture located on the eastern edge of the Qinghai–Tibet Plateau, Southwest China. Several vector-borne bacterial pathogens were screened and studied. *Anaplasma bovis* strains representing novel genotypes were detected in ticks (8.83%, 37/419), yak blood samples (45.71%, 64/140), and goat blood samples (58.93%, 33/56). Two spotted fever group (SFG) *Rickettsiae*, *Candidatus* Rickettsia jingxinensis, and a novel *Rickettsia* species named *Candidatus* Rickettsia hongyuanensis were identified in ticks. Another *Rickettsia* species closely related to the *Rickettsia* endosymbiont of *Polydesmus complanatus* was also detected in ticks. Furthermore, a *Coxiella* species was detected in ticks (3.34%, 14/419), keds (1.89%, 2/106), and yak blood (0.71%, 1/140). Interestingly, another *Coxiella* species and a *Coxiella*-like bacterium were detected in a tick and a goat blood sample, respectively. These results indicate the remarkable diversity of vector-borne pathogens circulating in this area. Further investigations on their pathogenicity to humans and domestic animals are still needed.

## 1. Introduction

Vector-borne diseases (VBDs) are diseases transmitted by arthropods, mainly by hematophagous arthropods such as ticks, mosquitoes, fleas, sand flies, lice, and mites. Vector-borne pathogens include bacteria (*Anaplasma*, *Rickettsia*, *Coxiella*, *Borrelia*, etc.), viruses (dengue virus, Crimean–Congo hemorrhagic fever virus, etc.), protozoans (*Plasmodium vivax*, *Leishmania donovani*, etc.), and even parasitic helminths (*Wuchereria bancrofti*, etc.) [1,2,3]. Due to the wide geographical distribution of hematophagous arthropods, globally emerging VBDs infect millions of people annually, especially in developing countries, and hence represent a significant threat to human health. Currently, vector-borne bacterial pathogens of most concern in China include *Rickettsia* spp., *Orientia* spp. (family Rickettsiaceae), *Anaplasma* spp. (family Anaplasmataceae), and *Coxiella* spp. (family Coxiellaceae) [4,5]. *Anaplasma phagocytophilum*, *A. bovis*, and spotted fever group *Rickettsia* have been frequently detected in ticks and animals from multiple provinces in China [6,7]. Cases of human infection with these pathogens have occasionally been reported [4,8]. Notably, domestic ungulates are often infected with *Anaplasma* spp., causing weight loss, abortion, reduced milk production, or even death, leading to numerous economic losses in the livestock industry annually [9]. Meanwhile, many infected domestic ungulates are permanently and asymptomatically infected. They may act as reservoirs and sources of pathogens for vectors in their life cycles. Meanwhile, *Coxiella burnetii*, the etiologic agent of Q fever, is the most notorious pathogen of the genus *Coxiella* [10]. As a worldwide distributed pathogen, it has been detected in multiple animal species, including goats, cattle, horses, deer, rodents, and even birds [11,12,13]. *Coxiella burnetii* infection in domestic animals is usually asymptomatic but may lead to infertility, abortion, and neonatal death [14], thus resulting in tremendous economic losses. Furthermore, human Q fever caused by *C. burnetii* is a worldwide zoonosis that has long been under-reported and underdiagnosed due to its non-specific symptoms.

Ngawa Tibetan and Qiang Autonomous Prefecture has an area of 84,242 km^2^. It is located in the northwest of Sichuan Province and on the eastern edge of the Qinghai–Tibet Plateau, China. The high altitude (982–6250 m, mostly 2500–4000 m) and the abundant grassland resources of Ngawa make animal husbandry the major income source for rural households. Yaks, goats, horses, and pigs are the common livestock animals in Ngawa. The frequent contact between herdsmen and livestock animals makes it possible for zoonotic and vector-borne pathogens to be easily transmitted from animals or their ectoparasites to humans. In recent years, some studies on vector-borne pathogens have been performed in some areas of the eastern Qinghai–Tibet Plateau [15,16,17]. In 2016, Yang et al. reported a novel *Anaplasma* species closely related to *A. capra* in *H. qinghaiensis* ticks from Gannan Tibetan Autonomous Prefecture located in the east Qinghai–Tibet Plateau [15]. In 2020, diverse *Bartonella* species were detected in *H. qinghaiensis* and *Dermacentor everestianus* ticks collected in Shiqu County, located on the Qinghai–Tibetan Plateau in Ganze Prefecture [16]. However, except for one study on *Theileria* detected in *H. qinghaiensis*, *D. everestianus*, *Rhipicephalus microplus*, and *Melophagus ovinus* reported in 2020 [17], vector-borne pathogens in Ngawa remain understudied.

The objective of our study was to update the epidemiological profile of circulating bacterial VBDs in Ngawa. In this study, we collected ticks (*H. qinghaiensis*), sheep keds (*Melophagus ovinus*), and the blood of yaks and goats in Hongyuan County of Ngawa. The presence, prevalence, and genetic characteristics of several vector-borne bacterial pathogens were studied.

## 2. Results

### 2.1. Sample Collection

**Figure 1:**
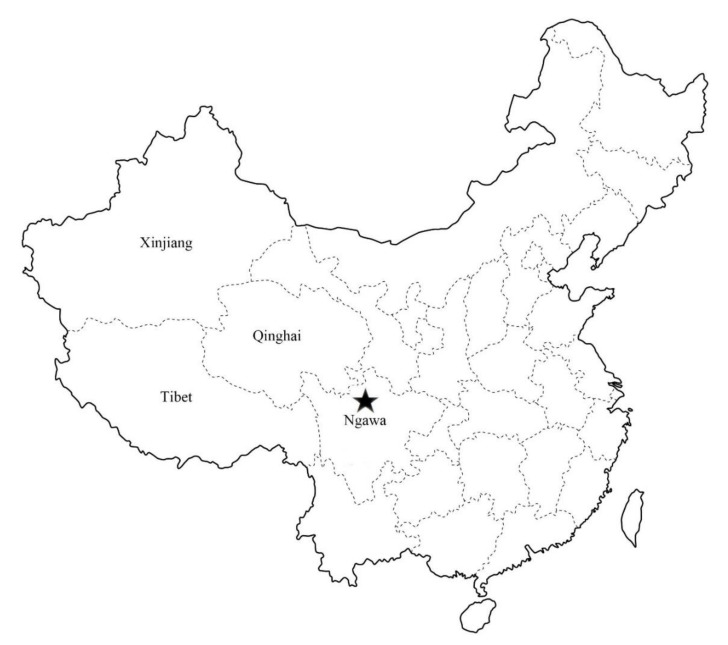
A map showing the location of Ngawa Tibetan and Qiang Autonomous Prefecture, Sichuan Province, China.

From February to March 2021, a total of 419 ticks (adults and nymphs) were collected from yaks (*Bos mutus*), and 106 keds were collected from goats (*Capra hircus*) in Hongyuan County of Ngawa, China (Figure 1). All ticks were morphologically determined to be *H. qinghaiensis* and further confirmed by analysis of the COI gene (accession numbers: ON358162-ON358175). All keds were morphologically identified as *M. ovinus* and molecularly confirmed (accession numbers: ON358236-ON358247). From March to May 2021, 140 and 56 blood samples were collected from apparently healthy yaks and goats, respectively.

### 2.2. PCR Detection

The PCR products of the 16S rDNA gene that met the expected lengths (*Rickettsia*: 900 bp; *Anaplasma*: 900 bp; *Coxiella*: 600 bp) were selected and subjected to sequencing. *Anaplasma bovis* was detected in 37 tick samples (8.83%, 37/419), 64 yak blood samples (45.71%, 64/140), and as many as 33 goat blood samples (58.93%, 33/56) (Table 1). All sequences of the 16S rDNA gene have the highest (99.21–100%) identity to *A. bovis* isolate Zhouzhi-goat-29 (MH255938.1) identified from goats in Shaanxi Province, China.

In total, three *Rickettsia* species, including *Candidatus* R. jingxinensis (0.48%, 2/419), SFG *Rickettsia* strain tick61 (0.24%, 1/419), and *Rickettsia* sp. strain tick14 (0.24%, 1/419), were identified in ticks (Table 1). Their 16S genes have the highest identities of 99.92% to *Candidatus* Rickettsia jingxinensis strains, 100% to uncultured *Rickettsia* sp. Hja 192 (LC379493.1) from *Ha. japonica* in Japan, and 99.83% to uncultured Rickettsiales bacterium clone LG127_1 (MK616427.1) from *Pompholyxophrys punicea*, respectively. Three *Coxiella* species were detected in these samples. A *Coxiella* strain closely related to *Coxiella* sp. isolate XinXian-HL9 (MG906671.1) identified from *H. longicornis* in China (with a sequence identity of 99.24–99.32% for the 16S gene) was detected in 14 of the tick samples (3.34%, 14/419), 2 of the ked samples (1.89%, 2/106), and 1 of the yak blood samples (0.71%, 1/140) (Table 1). *Coxiella* strain tick8, closely related to *Coxiella* endosymbionts, was detected in a tick sample with a 99.32% identity to the *Coxiella* endosymbiont of *Rhipicephalus turanicus* (JQ480818.1) and 99.24% identity to the *Coxiella* endosymbiont of *Rhipicephalus microplus* strain Porto Alegre (KY026064.1) and the *Coxiella* endosymbiont of *Rhipicephalus australis* isolate Rhaus2 (KP994830.1). Interestingly, *Coxiella*-like bacterium strain goat12 was identified from one goat blood sample, and the 16S rDNA shares the highest (96.77%) identity with *C. burnetii* strains (strain CB_25, strain CB_30, etc.).

### 2.3. Amplification of Key Genes and Genetic and Phylogenetic Analysis

**Figure 2:**
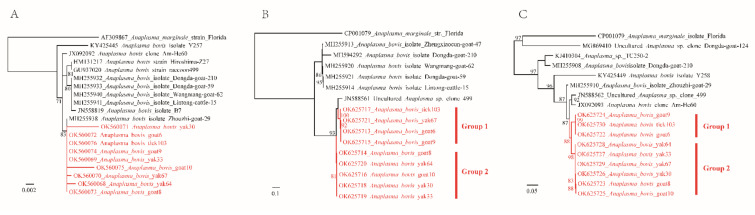
Phylogenetic trees constructed by the PhyML 3.0 software based on the nucleotide sequences of 16S rRNA (881 bp), *gltA* (826 bp), and *groEL* (769 bp) genes of *Anaplasma bovis* strains. (**A**): 16S rRNA, (**B**): *gltA*, (**C**): *groEL*.

For further characterization of the detected bacterial strains, sequences of *groEL* (769 bp) and *gltA* (826 bp) genes were recovered from randomly selected *A. bovis* strains from goats, yaks, and ticks. The *gltA* sequences have 92.23–92.74% identities to uncultured *Anaplasma* sp. clone 499 (JN588561.1) from raccoons in Japan. The *groEL* sequences have 95.54–96.49% similarities to *A. bovis* isolate Zhouzhi-goat-29 and 94.93–95.32% similarities to uncultured *Anaplasma* sp. clone 499 (JN588562.1) (Table S1). These results suggest that these strains are divergent from previously reported genotypes of *A. bovis*. As shown in the phylogenetic trees, the *gltA* and *groEL* genes are divided into two clusters (Figure 2). The *A. bovis* strains tick103, goat6, and goat9 belong to group 1, while strains goat8, goat10, yak30, and yak33 belong to group 2. Interestingly, the *gltA* gene of strain goat67 belongs to group 1, whereas its *groEL* gene is clustered in group 2.

**Figure 3:**
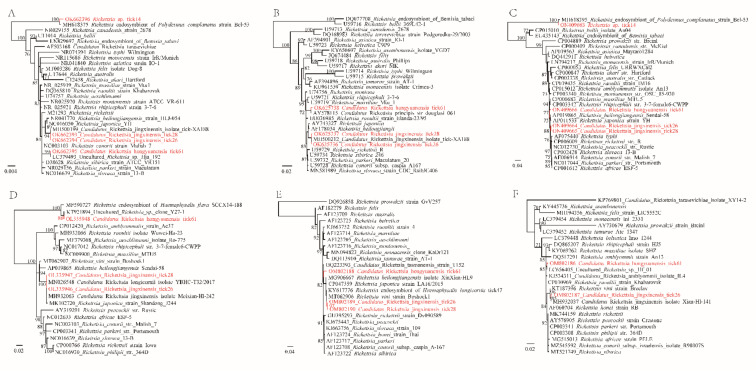
Phylogenetic trees constructed by the PhyML 3.0 software based on the nucleotide sequences of 16S rRNA (1201 bp), *gltA* (1006 bp), *groEL* (656–1053 bp), *ompA* (687–699 bp), *ompB* (376 bp), and *htrA* (411 bp) genes of *Rickettsia* strains. (**A**): 16S rRNA, (**B**): *gltA*, (**C**: *groEL*, (**D**): *ompA*, (**E**): *ompB*, (**F**): *htrA*.

A longer 16S fragment (1201 bp) and the *groEL* (656–1053 bp), *gltA* (1006 bp), *ompA* (705 bp), *ompB* (376 bp), and *htrA* (411 bp) genes were successfully recovered for the *Rickettsia* strains. The *groEL*, *gltA*, *ompA*, and *htrA* genes of the *Candidatus* R. jingxinensis strains have the highest identities of 99.05%, 100.00%, 100%, and 99.76% to other strains (*Candidatus* Rickettsia jingxinensis isolate Meixian-Hl-107, isolate tick-XA172, etc.). For the SFG *Rickettsia* strain tick61, although the 16S gene is 100% identical to *Rickettsia* sp. Hja 192 (LC379493.1) [18], its *groEL*, *gltA*, *ompA*, and *htrA* genes show 98.77%, 99.90%, 99.83%, 99.76% nucleotide similarity to *R. peacockii* str. Rustic (CP001227.1) from *Dermacentor andersoni* [19], *Candidatus* Rickettsia principis str. douglasi 061 (AY578115.1) from *Ha. japonica* [20], uncultured *Rickettsia* sp. clone Y27-1 (KT921894.1) from *H. qinghaiensis* [21], and uncultured *Rickettsia* sp. Hf_01 (LC656405) from *Ha. flava* in Japan, respectively (Table S2). As to the *ompB* gene, it has only as low as 97.07% (365/376 nt) nucleotide identity to *Candidatus* Rickettsia tasmanensis strain T152 (GQ223393) identified from *Ixodes tasmani* in Australia [22]. In the phylogenetic trees, its *groEL*, *gltA*, *ompA*, *ompB*, and *htrA* genes are apparently divergent from other SFG *Rickettsia* species (Figure 3). Notably, its *ompA* gene is in a basal location in the phylogenetic tree but far from SFG Rickettsia species. When only compared to validated *Rickettsia* species, its 16S, *gltA*, *groEL*, *ompA*, and *ompB* genes have the highest identities (99.58%, 98.81%, 98.77%, 81.87%, and 96.81%) to *R. conorii* strain Malish7 (NR074480) [23], *R. raoultii* strain Khabarovsk (CP010969) [24], *R. peacockii* str. Rustic (CP001227.1) [19], *R. japonica* strain YH_M (AP017602) [25], and *R. conorii* subsp. caspia A-167 (AF123708) [26], respectively. According to the criteria established by Fournier et al. [27], these results suggest that it represents a novel *Rickettsia* species. Based on the location where it was first characterized, we named it *Candidatus* R. hongyuanensis. For the *Rickettsia* sp. strain tick14, its *groEL* sequence has 99.54% (648/651 nt) nucleotide identity to the *Rickettsia* endosymbiont of *Polydesmus complanatus* strain Bel-53 (MH618395.1) identified in millipede [28] and 80.54% nucleotide identity to the *Rickettsia* endosymbiont of *Bemisia tabaci* (EU435143.1) identified in whitefly [29].

**Figure 4:**
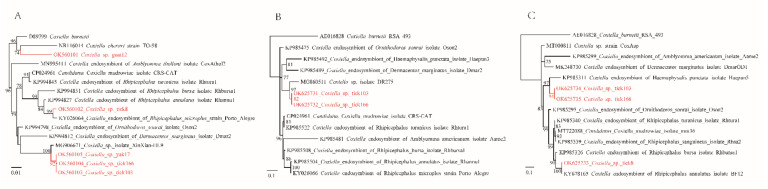
Phylogenetic trees constructed by the PhyML 3.0 software based on the nucleotide sequences of 16S rRNA (1180–1181 bp), *groEL* (545 bp), and *rpoB* (408–526 bp) genes of *Coxiella* strains. (**A**): 16S rRNA, (**B**): *groEL*, (**C**): *rpoB*.

*RpoB* (408–526 bp) and *groEL* (545 bp) genes were obtained from the *Coxiella* strains. For the *Coxiella* strain closely related to *Coxiella* sp. isolate XinXian-HL9, its *rpoB* sequence has 86.96% identity (400/460, 87% coverage) to *Coxiella* sp. strain CoxAsp (MT000811.1) from *Amblyomma* sp. and 83.88% identity (385/459, 86% coverage) to the *Coxiella* endosymbiont of *Dermacentor marginatus* isolate DmarOD1 (MK248730.1). Meanwhile, its *groEL* gene has the highest (91.56%) similarity (499/545 nt) to *Coxiella* sp. isolate DR275 (MG860511.1) from *Dermacentor reticulatus* in Slovakia (Table S3). As shown in the phylogenetic trees, its genes form distinct clusters from any other *Coxiella* species (Figure 4). For the *Coxiella* strain tick8, closely related to *Coxiella* endosymbionts, only the *rpoB* gene was obtained. The *rpoB* gene shows 99.51% similarity (403/405 nt) to the *Coxiella* endosymbiont of *Rhipicephalus annulatus* isolate BF12 (KY678165.1) and 98.77% similarity (400/405 nt) to the *Coxiella* endosymbiont of *Rhipicephalus microplus* strain Porto Alegre (KY026067.1). The *rpoB* and *groEL* genes were not recovered from the *Coxiella*-like bacterium detected in the goat sample.

All of these sequences have been submitted to Genbank (Shown in Table S4).

## 3. Discussion

In this study, most pathogens were detected in *H. qinghaiensis* ticks. Meanwhile, some pathogens detected in ticks were also detected in goats, yaks, and keds, indicating the possible role of *H. qinghaiensis* in the maintenance and transmission of these pathogens in this area. *Haemaphysalis qinghaiensis* is an endemic tick species widely distributed in the western plateau of China [15]. As a three-host tick, it often infests domestic animals, including goats, cattle, yaks, horses, etc. Occasionally, it also bites humans [15]. In previous studies, it has been demonstrated that *H. qinghaiensis* transmits a lot of human and animal pathogens, such as *Theileria* sp., *Babesia* sp., *Borrelia burgdorferi*, and *A. phagocytophilum* [21]. The potential role of *H. qinghaiensis* ticks in the transmission of human and animal diseases in this area should be further considered.

*Anaplasma bovis* has been well recognized as a tick-borne pathogen infecting cattle, buffalos, yaks, goats, and rodents, as well as humans [4,30,31,32]. It is one of the etiologic agents of bovine anaplasmosis, characterized by fluctuating fever, depression, and death [33]. Occasionally, it also infects humans and causes fever, headache, myalgia, rash, eschar, etc. [4]. In this study, we observed remarkably high positivity rates of *A. bovis* in apparently healthy goats (58.93%) and yaks (45.71%), indicating that *A. bovis* is circulating in domestic animals in this region. Genetic and phylogenetic analyses both indicate that these strains are different from previously reported *A. bovis* strains, and they may represent more than one novel genotype. Phylogenetic analysis based on *groEL* and *gltA* genes indicated that they are divided into two clusters, and genetic recombination between the two clusters may exist, suggesting the long-history evolution of *A. bovis* in this area. The pathogenicity of these strains to humans is still to be determined. Ticks also tested positive for this *A. bovis* strain, with a positivity rate of 8.83%. As reported previously, the detection rate of *Anaplasma* in ticks was likely skewed due to *Anaplasma* infection in livestock [33]. Although the possibility cannot be ruled out that the *A. bovis* DNA may come from the blood meal of ticks, we suspect that *H. qinghaiensis* ticks might play a role in the transmission of *A. bovis* in domestic animals.

Three *Rickettsia* species, including *Rickettsia* sp. strain tick14, *Candidatus* R. jingxinensis, and a novel SFG *Rickettsia* species named *Candidatus* R. hongyuanensis, were identified in ticks. *Candidatus* R. jingxinensis, belonging to SFG Rickettsia, was first identified in *Ha. longicornis* from northeast China in 2016 [34]. In recent years, it has been detected in *Rhipicephalus microplus*, *Ha. turturis*, and *Ixodes persulcatus* from multiple locations in China, Korea, Thailand, and India, indicating that it is a widely distributed *Rickettsia* circulating in Asia [35,36,37,38,39]. Notably, a *gltA* sequence (KU853023) from a patient in China was submitted to GenBank (unpublished), which shows 99.91% nucleotide identity to Ca. R. jingxinensis, suggesting its potential human pathogenicity. Furthermore, a novel SFG *Rickettsia* named *Candidatus* R. hongyuanensis was identified in *H. qinghaiensis* ticks. Its 16S, *gltA*, *groEL*, *ompA*, and *ompB* genes have the highest identities of 99.58%, 98.81%, 98.77%, 81.87%, and 96.81% to validated species, supporting that it represents a novel Rickettsia species based on criteria established by Fournier et al. [27]. Genetic analyses indicate that it is closely related to human pathogenic *R. raoultii* and *R. conorii.* Due to the fact that *H. qinghaiensis* is a three-host tick, it can easily transmit pathogens between different hosts during its life cycle [15]. Therefore, the role of *H. qinghaiensis* in the transmission of SFG *Rickettsia* should be further considered, and investigations on the human pathogenicity of these *Rickettsia* species are still needed. Furthermore, *Rickettsia* sp. strain tick14, closely related to the *Rickettsia* endosymbiont of *Polydesmus complanatus* Bel-53 [28], was identified, and it is located in a basic position of the phylogenetic trees based on 16S and *groEL* sequences.

Similar to Rickettsiales bacteria, the genus *Coxiella*, belonging to Coxiellaceae, is a group of intracellular bacteria [40]. This genus includes two recognized species (*C. burnetii* and *C. cheraxi*), one Candidatus species (*Candidatus* Coxiella mudrowiae), and some unclassified *Coxiella* symbionts [41,42]. In this study, three *Coxiella* species were detected, with two of them infecting domestic animals. This may be one of the few recent studies in which *Coxiella* species were found to infect mammals. One *Coxiella* species (strain yak17, strain tick103, and strain tick166) was detected in ticks (3.34%), sheep keds (1.89%), and one yak blood sample (0.71%). Interestingly, sheep keds also tested positive for this *Coxiella*. There might be three possibilities: (1). Sheep keds are infected, and they act as the vector of this *Coxiella*. (2). The *Coxiella* DNA is from the blood meal of sheep keds. That is, goats may be infected by this *Coxiella*. (3). The *Coxiella* strains could be just *Coxiella*-like endosymbionts that co-exist with hosts/vectors. Each possibility is intriguing, and further studies are still needed. Furthermore, it is noteworthy that a *Coxiella*-like bacterium was identified in a goat blood sample. Its 16S gene shared the highest (96.77%) identity with the human and animal pathogen *C. burnetii* strain CB_25 (LC464975.1), strain CB_30 (LC464974.1), etc. (unpublished). However, sequences of other key genes are not available for this strain, possibly due to the low identity to known strains.

In summary, we identified seven bacterial species in arthropods and domestic animals in Ngawa. Although the human pathogenicity for most of these bacteria is still to be determined, more attention should be paid to the risk of human infection and the possible circulation of these pathogens in local people.

## 4. Materials and Methods

### 4.1. Sample Collection and Processing

Ngawa Tibetan and Qiang Autonomous Prefecture is located in the northwest of Sichuan Province and on the eastern edge of Qinghai–Tibet Plateau, China. From February to March 2021, 419 ticks and 106 sheep keds were respectively collected from yaks and goats in Sedi town (102.98°E, 30.01°N, with an altitude of 3528 m), Hongyuan County of Ngawa, Sichuan Province, China. The ticks and keds were stored at −40 °C before DNA extraction. From March to May, blood samples were collected from asymptomatic domestic animals, including yaks and goats, in Hongyuan County. Whole blood samples were stored in tubes containing EDTA as anticoagulant and then stored at −40 °C before DNA extraction.

### 4.2. DNA Extraction

The cryopreserved tick and sheep ked samples were washed three times with phosphate-buffered saline (PBS) and then were individually placed into 2 mL sterile tubes with 0.5 mL of PBS solution. After homogenization using Mixer Mill MM 400 (Retsch, Hann, Germany), the arthropod suspension was directly subjected to DNA extraction with a Mollusc DNA Extraction Kit (Omega Bio-Tek, Norcross, GA, USA). The kit was operated according to the manufacturer’s instructions, and the genomic DNA was extracted and eluted with 60 μL of Elution Buffer twice.

According to the protocol of Blood & Tissue DNA Kit (Omega, Norcross, GA, USA), DNA was extracted from 200 μL of the animal blood samples. After extraction, the DNA samples were diluted into 60 μL of Elution Buffer repeatedly.

### 4.3. Species Identification of Ticks and Keds

Ticks and keds were morphologically identified by an arthropod taxonomist using microscopy and then stored at −40 °C until DNA isolation. The tick species was morphologically determined by observing the basis scapituli and palp, while the ked species was determined by checking the mouthparts, bristles on the body surface, and legs tipped with pointed claws [43]. Tick and ked species were further confirmed by PCR amplification and analysis of the COI gene (cytochrome C oxidase I) (primers shown in Table S5). PCR amplification was performed using a Sensoquest PCR System LabCycler (Göttingen, Germany). The cycling conditions were: 94 °C for 3 min, 35 cycles of 94 °C for 30 s, 55 °C for 1 min, 72 °C for 1 min, and a final extension of 72 °C for 5 min [44]. After electrophoresis, the amplicons were subjected to sequencing and analyzed by BLASTN alignment.

### 4.4. PCR Detection and Amplification of Key Genes

To determine the presence and prevalence of *Rickettsia* spp., *Anaplasma* spp., and *Coxiella* spp., semi-nested or nested PCR targeting a conserved domain of 16S rDNA gene was performed on all DNA samples. The primers are shown in Table S5 and previous reports [43,45]. PCR amplification was performed using a Sensoquest PCR System LabCycler (Germany). For further analysis of the molecular characteristics and phylogenetic positions of these bacterial strains, the sequences of two other genes (the *gltA* gene encoding citrate synthase and the *groEL* gene encoding the heat shock protein) were obtained for *Rickettsia* (*gltA*: 1006 bp; *groEL*: 656–1053 bp) and *Anaplasma* (*gltA*: 826 bp; *groEL*: 769 bp) positive samples (primers and details shown in Table S5). Additionally, a longer 16S fragment (1201 bp), *ompA* (705 bp), *ompB* (376 bp), and *htrA* (411 bp) genes were recovered from *Rickettsia* strains. The primers used were previously described [43,46] (Table S5). For *Coxiella* strains, the sequences of *groEL* and *rpoB* genes were obtained using the primers shown in Table S5 [45]. The details of amplification are provided in Table S5. After electrophoresis in 1.0% agarose gel, the PCR amplicons were subjected to sequencing by Sangon Biotech Company (Shanghai, China), and the sequencing results were analyzed by BLASTN alignment.

### 4.5. Genetic and Phylogenetic Analyses

Protein-coding sequences from *Rickettsia* spp., *Anaplasma* spp., and *Coxiella* spp. obtained in this study, as well as the reference sequences retrieved from GenBank, were aligned using ClustalW (“protein-coding genes” strategy) in the MEGA software, version 7.0 [47]. Nucleotide sequence identities were calculated by MegAlign program in the DNASTAR Lasergene package (DNASTAR, Inc., Madison, WI, USA). Phylogenetic trees of the 16S and other key genes based on the maximum likelihood method were reconstructed by PhyML v3.2 [48]. The substitution model test was performed to determine the best-fit phylogenetic model. The confidence values for individual branches of the tree were determined by bootstrap analysis with 100 repetitions.

## Figures and Tables

**Table 1:** The positivity rates of *Anaplasma*, *Rickettsia*, and *Coxiella* pathogens in different hosts (ticks, keds, yaks, and goats).

Bacterial Species	*Haemaphysalis qinghaiensis*	*Melophagus ovinus*	*Bos mutus*	*Capra hircus*
*Anaplasma bovis*	8.83% (37/419)	0.00% (0/106)	45.71% (64/140)	58.93% (33/56)
*Candidatus* Rickettsia jingxinensis	0.48% (2/419)	0.00% (0/106)	0.00% (0/140)	0.00% (0/56)
*Candidatus* Rickettsia hongyuanensis	0.24% (1/419)	0.00% (0/106)	0.00% (0/140)	0.00% (0/56)
*Rickettsia* sp. tick14	0.24% (1/419)	0.00% (0/106)	0.00% (0/140)	0.00% (0/56)
*Coxiella* spp.	3.34% (14/419)	1.89% (2/106)	0.71% (1/140)	0.00% (0/56)
*Coxiella* sp. tick8	0.24% (1/419)	0.00% (0/106)	0.00% (0/140)	0.00% (0/56)
*Coxiella*-like bacterium	0.00% (0/419)	0.00% (0/106)	0.00% (0/140)	1.79% (1/56)
Total	13.37% (56/419)	1.89% (2/106)	46.43% (65/140)	60.71% (34/56)

## Data Availability

All sequence files are available from the NCBI database (shown in Table S4).

## Supplementary Materials

The following supporting information can be downloaded at: https://www.mdpi.com/article/10.3390/pathogens11050606/s1, Table S1. Nucleotide identity of 16S, *gltA*, and *groEL* genes of *Anaplasma bovis* strains to reported strains in the Genbank database. Table S2. Nucleotide identity of 16S, *gltA*, *groEL*, *ompA*, *ompB*, and *htrA* genes of *Rickettsia* strains to reported strains in the Genbank database. Table S3. Nucleotide identity of 16S, *groEL*, and *rpoB* genes of *Coxiella* strains to reported strains in the Genbank database. Table S4. Genbank numbers of *Rickettsia* spp., *Anaplasma* spp., and *Coxiella* spp. sequences obtained in this study. Table S5. The primers used for amplification of 16S, *gltA*, *groEL*, *htrA*, *ompA*, *ompB*, and *rpoB* genes from *Rickettsia* spp., *Anaplasma* spp., and *Coxiella* spp. by nested PCR or semi-nested PCR. Supporting Data. Nucleotide sequences of *Candidatus* Rickettsia hongyuanensis.
