# Supplementary material for: Molecular Survey of Vector-Borne Pathogens in Ticks, Sheep Keds, and Domestic Animals from Ngawa, Southwest China
Source: Pathogens. 2022 May 22;11(5):606. doi: 10.3390/pathogens11050606 (PMC9143929; doi:10.3390/pathogens11050606)
Supplement: Supplementary file 1 [file pathogens-11-00606-s001.zip › Table S1.pdf]

Table S1. Nucleotide identity of 16S, *gltA*, and *groEL* genes of *Anaplasma bovis* strains to reported strains in the Genbank database.

	16S	*gltA*	*groEL*
Anaplasma bovis goat6	100%<br>*Anaplasma bovis* isolate Zhouzhi-goat-29	92.23%<br>Uncultured *Anaplasma* sp. clone 499	96.49%<br>*Anaplasma bovis* isolate Zhouzhi-goat-29
Anaplasma bovis goat8	100%<br>*Anaplasma bovis* isolate Zhouzhi-goat-29	92.37%<br>Uncultured *Anaplasma* sp. clone 499	95.54%<br>*Anaplasma bovis* isolate Zhouzhi-goat-29
Anaplasma bovis goat9	100%<br>*Anaplasma bovis* isolate Zhouzhi-goat-29	92.23%<br>Uncultured *Anaplasma* sp. clone 499	96.49%<br>*Anaplasma bovis* isolate Zhouzhi-goat-29
Anaplasma bovis goat10	99.32%<br>*Anaplasma bovis* isolate Zhouzhi-goat-29	92.48%<br>Uncultured *Anaplasma* sp. clone 499	95.54%<br>*Anaplasma bovis* isolate Zhouzhi-goat-29
Anaplasma bovis yak30	99.21%<br>*Anaplasma bovis* isolate Zhouzhi-goat-29	92.74%<br>Uncultured *Anaplasma* sp. clone 499	95.68%<br>*Anaplasma bovis* isolate Zhouzhi-goat-29
Anaplasma bovis yak33	100%<br>*Anaplasma bovis* isolate Zhouzhi-goat-29	92.74%<br>Uncultured *Anaplasma* sp. clone 499	95.81%<br>*Anaplasma bovis* isolate Zhouzhi-goat-29
Anaplasma bovis yak64	99.77%<br>*Anaplasma bovis* isolate Zhouzhi-goat-29	92.74%<br>Uncultured *Anaplasma* sp. clone 499	95.81%<br>*Anaplasma bovis* isolate Zhouzhi-goat-29
Anaplasma bovis yak67	99.89%<br>*Anaplasma bovis* isolate Zhouzhi-goat-29	92.23%<br>Uncultured *Anaplasma* sp. clone 499	95.81%<br>*Anaplasma bovis* isolate Zhouzhi-goat-29
Anaplasma bovis tick103	100%<br>*Anaplasma bovis* isolate Zhouzhi-goat-29	92.48%<br>Uncultured *Anaplasma* sp. clone 499	96.49%<br>*Anaplasma bovis* isolate Zhouzhi-goat-29
