# Supplementary material for: Molecular Survey of Vector-Borne Pathogens in Ticks, Sheep Keds, and Domestic Animals from Ngawa, Southwest China
Source: Pathogens. 2022 May 22;11(5):606. doi: 10.3390/pathogens11050606 (PMC9143929; doi:10.3390/pathogens11050606)
Supplement: Supplementary file 1 [file pathogens-11-00606-s001.zip › Table S2.pdf]

Table S2. Nucleotide identity of 16S, *gltA*, *groEL*, *ompA*, *ompB*, and *htrA* genes of *Rickettsia* strains to reported strains in the Genbank database.

	16S	*gltA*	*groEL*	*ompA*	*ompB*	*htrA*
Ca. Rickettsia hongyuanensis tick61	100% uncultured *Rickettsia* sp. Hja 192	99.90% *Ca.* Rickettsia principis str. douglasi 061	98.77% *Rickettsia peacockii* str. Rustic	99.83% Uncultured *Rickettsia* sp. clone Y27-1	97.07% *Ca.* Rickettsia tasmanensis str. T152	99.76% Uncultured *Rickettsia* sp. Hf_01
Ca. Rickettsia jingxinensis tick26	99.92% Ca. *Rickettsia* jingxinensis	100% *Ca.* Rickettsia jingxinensis	99.05% *Rickettsia heilongjiangensis*	100% *Ca.* Rickettsia jingxinensis	99.20% *Rickettsia* sp. strain Koreansis	99.76% *Ca.* Rickettsia jingxinensis
Ca. Rickettsia jingxinensis tick28	99.92% Ca. *Rickettsia* jingxinensis	100% *Ca.* Rickettsia jingxinensis	99.05% *Rickettsia heilongjiangensis*	100% *Ca.* Rickettsia jingxinensis	98.94% *Rickettsia* sp. strain Koreansis	NA
Rickettsia sp. tick14	99.83% Uncultured Rickettsiales bacterium clone LG127_1	NA	99.54% *Rickettsia* endosymbiont of *Polydesmus complanatus* Bel-53	NA	NA	NA
