# Supplementary material for: Molecular Survey of Vector-Borne Pathogens in Ticks, Sheep Keds, and Domestic Animals from Ngawa, Southwest China
Source: Pathogens. 2022 May 22;11(5):606. doi: 10.3390/pathogens11050606 (PMC9143929; doi:10.3390/pathogens11050606)
Supplement: Supplementary file 1 [file pathogens-11-00606-s001.zip › Table S3.pdf]

Table S3. Nucleotide identity of 16S, *groEL* and *rpoB* genes of *Coxiella* strains to reported strains in the Genbank database.

	16S	*groEL*	*rpoB*
Coxiella sp. yak17	99.32%<br>*Coxiella* sp. isolate XinXian-HL9	NA	NA
Coxiella sp. tick103	99.32%<br>*Coxiella* sp. isolate XinXian-HL9	91.56%<br>Coxiella sp. isolate DR275	86.96%<br>*Coxiella* sp. strain CoxAsp
Coxiella sp. tick166	99.24%<br>*Coxiella* sp. isolate XinXian-HL9	91.56%<br>Coxiella sp. isolate DR275	86.96%<br>*Coxiella* sp. strain CoxAsp
Coxiella sp. tick8	99.32%<br>*Coxiella* endosymbiont of *Rhipicephalus turanicus*	NA	99.51%<br>*Coxiella* endosymbiont of *Rhipicephalus turanicus*
Coxiella-like bacterium goat12	96.77%<br>*Coxiella burnetii*	NA	NA
