# Supplementary material for: Molecular Survey of Vector-Borne Pathogens in Ticks, Sheep Keds, and Domestic Animals from Ngawa, Southwest China
Source: Pathogens. 2022 May 22;11(5):606. doi: 10.3390/pathogens11050606 (PMC9143929; doi:10.3390/pathogens11050606)
Supplement: Supplementary file 1 [file pathogens-11-00606-s001.zip › Table S4.pdf]

Table S4 Genbank numbers of *Rickettsia*, *Anaplasma*, and *Coxiella* sequences obtained in this study.

	Gene	Genbank numbers	Bacterial strain
1	16S	OK560068	*Anaplasma_bovis*_yak64
2	16S	OK560069	*Anaplasma_bovis*_yak33
3	16S	OK560070	*Anaplasma_bovis*_yak67
4	16S	OK560071	*Anaplasma_bovis*_yak30
5	16S	OK560072	*Anaplasma_bovis*_goat6
6	16S	OK560073	*Anaplasma_bovis*_goat8
7	16S	OK560074	*Anaplasma_bovis*_goat9
8	16S	OK560075	*Anaplasma_bovis*_goat10
9	16S	OK560076	*Anaplasma_bovis*_tick103
10	16S	OK560101	*Coxiella*-like_bacterium_goat12
11	16S	OK560102	*Coxiella*_sp._tick8
12	16S	OK560103	*Coxiella*_sp._tick103
13	16S	OK560104	*Coxiella*_sp._tick166
14	16S	OK560105	*Coxiella*_sp._yak17
15	16S	OK662394	*Candidatus* _Rickettsia_jingxinensis_tick26
16	16S	OK662393	*Candidatus* _Rickettsia_jingxinensis_tick28
17	16S	OK662395	*Candidatus* _Rickettsia_hongyuanensis_tick61
18	16S	OK662396	*Rickettsia*_sp._tick14
19	*gltA*	OK625713	*Anaplasma_bovis*_goat6
20	*gltA*	OK625714	*Anaplasma_bovis*_goat8
21	*gltA*	OK625715	*Anaplasma_bovis*_goat9
22	*gltA*	OK625716	*Anaplasma_bovis*_goat10
23	*gltA*	OK625717	*Anaplasma_bovis*_tick103
24	*gltA*	OK625718	*Anaplasma_bovis*_yak30
25	*gltA*	OK625719	*Anaplasma_bovis*_yak33
26	*gltA*	OK625720	*Anaplasma_bovis*_yak64
27	*gltA*	OK625721	*Anaplasma_bovis*_yak67
28	*gltA*	OK625736	*Candidatus* _Rickettsia_jingxinensis_tick26
29	*gltA*	OK625737	*Candidatus* _Rickettsia_jingxinensis_tick28
30	*gltA*	OK625738	*Candidatus* _Rickettsia_hongyuanensis_tick61
31	*groEL*	OK625722	*Anaplasma_bovis*_goat6
32	*groEL*	OK625723	*Anaplasma_bovis*_goat8
33	*groEL*	OK625724	*Anaplasma_bovis*_goat9
34	*groEL*	OK625725	*Anaplasma_bovis*_goat10
35	*groEL*	OK625726	*Anaplasma_bovis*_yak30
36	*groEL*	OK625727	*Anaplasma_bovis*_yak33
37	*groEL*	OK625728	*Anaplasma_bovis*_yak64
38	*groEL*	OK625729	*Anaplasma_bovis*_yak67

39	*groEL*	OK625730	*Anaplasma_bovis*_tick103
40	*groEL*	ON409664	*Candidatus* _Rickettsia_jingxinensis_tick26
41	*groEL*	ON409665	*Candidatus* _Rickettsia_jingxinensis_tick28
42	*groEL*	ON409666	*Candidatus* _Rickettsia_hongyuanensis_tick61
43	*groEL*	ON409663	*Rickettsia*_sp._tick14
44	*groEL*	OK625731	*Coxiella*_sp._tick103
45	*groEL*	OK625732	*Coxiella*_sp._tick166
46	*rpoB*	OK625733	*Coxiella*_sp._tick8
47	*rpoB*	OK625734	*Coxiella*_sp._tick103
48	*rpoB*	OK625735	*Coxiella*_sp._tick166
49	*ompA*	OL335946	*Candidatus* _Rickettsia_jingxinensis_tick26
50	*ompA*	OL335947	*Candidatus* _Rickettsia_jingxinensis_tick28
51	*ompA*	OL335948	*Candidatus* _Rickettsia_hongyuanensis_tick61
52	*htrA*	OM802186	*Candidatus*_Rickettsia_hongyuanensis_tick61
53	*htrA*	OM802187	*Candidatus*_Rickettsia_jingxinensis_tick26
54	*ompB*	OM802188	*Candidatus*_Rickettsia_hongyuanensis_tick61
55	*ompB*	OM802189	*Candidatus*_Rickettsia_jingxinensis_tick26
56	*ompB*	OM802190	*Candidatus*_Rickettsia_jingxinensis_tick28
