# Supplementary material for: Molecular Survey of Vector-Borne Pathogens in Ticks, Sheep Keds, and Domestic Animals from Ngawa, Southwest China
Source: Pathogens. 2022 May 22;11(5):606. doi: 10.3390/pathogens11050606 (PMC9143929; doi:10.3390/pathogens11050606)
Supplement: Supplementary file 1 [file pathogens-11-00606-s001.zip › Table S5.pdf]

Table S5 The primers used for amplification of 16S, *gltA*, *groEL*, *htrA*, *ompA*, *ompB*, *rpoB*, and *COI* genes from *Rickettsia*, *Anaplasma*, *Coxiella* and hosts by PCR.

Primer	Cycle	Bacteria/Host	Gene	annealing temperature	Sequence	Anticipated amplicon length	Reference
Ana-F	1, 2	*Anaplasma*	16S	52ºC	5-GATAGCCACTRGAAGTGGT-3	900 bp	Lu et al., 2022 (for detection)
Ana-R1	1	*Anaplasma*	16S	52ºC	5-CGTGCTGACTTGACATCAT-3		
Ana-R2	2	*Anaplasma*	16S	52ºC	5-CATCTCACGACACGAGCTG-3		
Ric-F	1, 2	*Rickettsia*	16S	50ºC	5-YTACGGAATAACTTTTAGAAA-3	900 bp	Lu et al., 2022 (for detection)
Ric-R1	1	*Rickettsia*	16S	50ºC	5-CATGATGACTTGACRTCGT-3		
Ric-R2	2	*Rickettsia*	16S	50ºC	5-CATCTCACGACACGAGCTG-3		
Cox-R	1, 2	*Coxiella*	16S	52ºC	5-ACTYYCCAACAGCTAGTTCTCA-3	600 bp	Duron et al., 2015 (for detection)
Cox-F1	1	*Coxiella*	16S	52ºC	5-GTAGGAATCTACCTTRTAGWGG-3		
Cox-F2	2	*Coxiella*	16S	52ºC	5-TGAGAACTAGCTGTTGGRGAGT-3		
Ric-glt-F1	1	*Rickettsia*	*gltA*	52ºC	5-ACTTAYGAYCCGGGCTTTAT-3	1100 bp	Lu et al., 2022
Ric-glt-F2	2	*Rickettsia*	*gltA*	52ºC	5-CTTTATGTCTACTGCTTCTTG-3		
Ric-glt-R	1, 2	*Rickettsia*	*gltA*	52ºC	5-AGCTGTCTAGGTCTGCTGATT-3		

Ric-gro-F1	1	*Rickettsia*	*groEL*	52ºC	5-CCATTACATGATAGAATTGCAA-3	800 bp	Lu et al., 2022
Ric-gro-F2	2	*Rickettsia*	*groEL*	52ºC	5-GAATTGCAATAAAGCCTATCG-3		
Ric-gro-R	1, 2	*Rickettsia*	*groEL*	52ºC	5-CCATCATTGCTTTTCTTCTATC-3		
Ana-glt-F1	1	*Anaplasma*	*gltA*	50ºC	5-CATCCNATGGCTATTYTCAT-3	900 bp	Lu et al., 2022
Ana-glt-R1	1	*Anaplasma*	*gltA*	50ºC	5-ACTATACCKGAGTAAAAGTC-3		
Ana-glt-F2	2	*Anaplasma*	*gltA*	50ºC	5-GAYCACGARCARAATGCTTC-3		
Ana-glt-R2	2	*Anaplasma*	*gltA*	50ºC	5-GAGTAAAAGTCGACRTTKGG-3		
Ana-gro-F1	1	*Anaplasma*	*groEL*	54ºC	5-GYCAGTGGGCTGGTAATGAA-3	1200 bp	Lu et al., 2022
Ana-gro-R1	1	*Anaplasma*	*groEL*	54ºC	5-CCWCCTGGTACWACACCTTC-3		
Ana-gro-F2	2	*Anaplasma*	*groEL*	52ºC	5-ATAGTYATGAAGGAGAGTGAT-3		
Ana-gro-R2	2	*Anaplasma*	*groEL*	52ºC	5-TCAACAGCAGCTCTAGTWG-3		
Cox-rpoB-F1	1	*Coxiella*	*rpoB*	56ºC	5-GGGCGRCAYGGWAAGAAAGGSGT-3	550 bp	Duron et al., 2015
Cox-rpoB-R1	1	*Coxiella*	*rpoB*	56ºC	5-CACCRAACCGTTGACCRCCAAATTG-3		
Cox-rpoB-F2	2	*Coxiella*	*rpoB*	56ºC	5-TCGAAGACATGCCYTATTTAGAAG-3		
Cox-rpoB-R2	2	*Coxiella*	*rpoB*	56ºC	5-AGCTTTCCCACCGARGGGTTGCTG-3		
Cox-gro-F1	1	*Coxiella*	*groEL*	56ºC	5-TTTGAAAAYATGGGCGCKCAAATGGT-3	600 bp	Duron et al., 2015
Cox-gro-F2	2	*Coxiella*	*groEL*	56ºC	5-GAAGTGGCTTCGCRTACWTCAGACG-3		

Cox-gro-R1	1	*Coxiella*	*groEL*	56ºC	5-CCAAACCCAGGTGCTTTYAC-3		
Cox-gro-R2	2	*Coxiella*	*groEL*	56ºC	5-CGRTCGCCAAAGCCAGGTGC-3		
QD8rpoBin5	1, 2	*Coxiella*-str.tick8	*rpoB*	51 ºC	5-GAYWTCGTRCTWAAYCCYTTAGG-3	400 bp	This study
QD8rpoBmid3	1	*Coxiella*-str.tick8	*rpoB*	51ºC	5-AWCCYGTGGARCGMGCGTGCAT-3		
QD8rpoBin3	2	*Coxiella*-str.tick8	*rpoB*	51ºC	5-TCATCTACTARRTGRTTAAGCTT-3		
QR14ELex5	1	*Rickettsia*-str.tick14	*groEL*	52ºC	5-ARRCACGGYTCARAWGCWCGT-3	650 bp	This study
QR14gELin5	2	*Rickettsia*-str.tick14	*groEL*	52ºC	5-ATRCCGTAAARGTTACWTTAGG-3		
QR14groEL3	1, 2	*Rickettsia*-str.tick14	*groEL*	52ºC	5-CCACCACGTAATYTATTKACYAC-3		
htrAex5	1	*Rickettsia*	*htrA*	53ºC	5-GCTTTACAAAATTCTAAAAACCATATA-3	400 bp	Borsoi et al., 2019
htrAex3	1	*Rickettsia*	*htrA*	53ºC	5-TGTCTATCAATTCACAACTTGC-3		
htrAin5	2	*Rickettsia*	*htrA*	48ºC	5-GCTCTTGCAACTTCTATGT-3		
htrAin5	2	*Rickettsia*	*htrA*	48ºC	5-CATTGTTCGTCAGGTTGGC-3		
ompBex5	1	*Rickettsia*	*ompB*	54ºC	5-GTAACCGGAAGTAATCGTTTCGTAA-3	400 bp	Borsoi et al., 2019
ompBex3	1	*Rickettsia*	*ompB*	54ºC	5-CTTTATAACCAGCTAAACCAC-3		
ompBin5	2	*Rickettsia*	*ompB*	51ºC	5-GTTTAATACGTGCTGCTAACCA-3		
ompBin3	2	*Rickettsia*	*ompB*	51ºC	5-GGTTTGGCCCATATACCATAA-3		
LCO1490	1	arthropods	*COI*	52ºC	5-GGTCAACAAATCATAAAGATATTGG-3	650 bp	Lu et al., 2022

HCO2198	1	arthropods	*COI*	52ºC	5-TAAACTTCAGGGTGACCAAAAAATCA-3
